# Is it reasonable to predict weaning by measuring diaphragm activity under ultrasound especially its reduction of excursion?

**DOI:** 10.1186/s13054-023-04585-5

**Published:** 2023-08-07

**Authors:** Linli Wang, Yaxiaerjiang Muhetaer, Ling Zhu, Qian Wang, Wei Wu

**Affiliations:** 1 Department of Critical Care Medicine, Zhongshan Hospital Affiliated to Fudan University, 180 Fenglin Road, Shanghai, China; 2 Emergency Department, Easthospital Affiliated to Tongji University, 1800 Yuntai Road, Shanghai, China

We read with interest the article by Daozheng Huang et al. entitled “Using automatic speckle tracking imaging to measure diaphragm excursion and predict the outcome of mechanical ventilation weaning” [1] and the correspondence “‘Under pressure’: should we use diaphragm excursion to predict weaning success in patients receiving pressure support ventilation?” by Emma Sabourin1 et al., and about this topic, we still have our own additional and important opinions.

Extensive existing researches revealed that diagram ultrasound plays important role in predicting weaning success or failure. And in the study of Daozheng Huang et al., they found that diaphragm excursion was of high diagnostic values for prolonged weaning and weaning failure in patients with invasive mechanical ventilation with support set at 10–12 cm H_2_O.

However, in Emma Sabourin’s correspondence, they pointed out that diaphragm excursion under pressure support mode could not be interpreted as the patient’s own respiratory muscle strength. They further suggested that in trials evaluating the prediction of weaning success in patients under pressure support, researchers should take diaphragmatic thickening fraction as the variable, which we quite agree.

Beside all these, however, we still firmly believe that it is crucial to re-examine the predictive value of diaphragm activity under ultrasound for predicting weaning.

First, in Daozheng Huang’s and other researches evaluating the predictive value of diaphragm activity for weaning, only reduction of diaphragm excursion was defined as diaphragm dysfunction which predicted weaning failure; actually, however, enhancement of diaphragm excursion also means weaning failure.

It is known that derangements of respiratory drive usually exist in critical patients. Situations such as systemic inflammation and pneumonia usually lead to excess loading of the diaphragm, which is manifested as excessive excursion of diaphragm [2], and exactly these patients usually require longer and higher supporting level of ventilation duration. Actually, Goligher had already illustrated that excess inspiratory effort measured by ultrasound meant worse clinical outcomes including prolonged ventilation, which meant failing to weaning normally [3]. And only intermediate diaphragm thickening fraction similar to healthy patients at rest (15–30%) was associated with shorter duration of ventilation, lower risk of reintubation and tracheostomy [3]. This is because in the absence of respiratory weakness, breathing effort arousing by respiratory drive determines the magnitude and frequency of respiratory muscles including diagram [4]. So we can make it clear that lots of diseases usually lead to excess respiratory drive and then arouses excess respiratory effort, which is presented as excess diaphragm activity measured by ultrasound, which can also indicate weaning failure.

So it is one-sided to use only reduction of diagram excursion regardless of its enhancement to predict weaning failure.

Last but not least, we emphasize here that weaning is a complex procedure and diaphragmatic activity should not be the only factor to be assessed. We should remember that measurements of diaphragm for weaning by ultrasound alone may lead to ignoring other factors associated with weaning failure and thus arousing inappropriate therapy decisions. There are more pathophysiological factors leading to weaning failure which should be taken into consideration, such as respiratory center dysfunction, respiratory muscles overload, cardiovascular dysfunction, or reduced airway protection and self-cleaning ability [5].

Sum up, diaphragm excursion measurement using speckle tracking imaging is an interesting approach, but it is one-sided and not rigorous to predict weaning success or failure only depends on reduction of diaphragm excursion while regardless of its enhancement. From a more comprehensive and scientific perspective, lots of other factors responsible for predicting weaning success or failure should be assessed together rather than the activity of diaphragm measurements by ultrasound solo. Further research on diaphragm function assessment together with other factors is required to predict weaning outcome.

## Data Availability

Not applicable.
